# A Web-Based Adaptation of the Quality of Life in Bipolar Disorder Questionnaire: Psychometric Evaluation Study

**DOI:** 10.2196/17497

**Published:** 2020-04-27

**Authors:** Emma Morton, Sharon HJ Hou, Oonagh Fogarty, Greg Murray, Steven Barnes, Colin Depp, CREST.BD, Erin Michalak

**Affiliations:** 1 Department of Psychiatry University of British Columbia Vancouver, BC Canada; 2 Faculty of Health, Arts and Design Swinburne University Hawthorn Australia; 3 University of Guelph Guelph, ON Canada; 4 Faculty of Medicine University of British Columbia Vancouver, BC Canada; 5 Department of Psychology University of British Columbia Vancouver, BC Canada; 6 Department of Psychiatry University of California San Diego, CA United States

**Keywords:** bipolar disorder, survey methodology, patient reported outcomes, psychometrics, questionnaire design, quality of life, validation studies

## Abstract

**Background:**

Quality of life (QoL) is considered a key treatment outcome in bipolar disorder (BD) across research, clinical, and self-management contexts. Web-based assessment of patient-reported outcomes offer numerous pragmatic benefits but require validation to ensure measurement equivalency. A web-based version of the Quality of Life in Bipolar Disorder (QoL.BD) questionnaire was developed (QoL Tool).

**Objective:**

This study aimed to evaluate the psychometric properties of a web-based QoL self-report questionnaire for BD (QoL Tool). Key aims were to (1) characterize the QoL of the sample using the QoL Tool, (2) evaluate the internal consistency of the web-based measure, and (3) determine whether the factor structure of the original version of the QoL.BD instrument was replicated in the web-based instrument.

**Methods:**

Community-based participatory research methods were used to inform the development of a web-based adaptation of the QoL.BD instrument. Individuals with BD who registered for an account with the QoL Tool were able to opt in to sharing their data for research purposes. The distribution of scores and internal consistency estimates, as indicated by Cronbach alpha, were inspected. An exploratory factor analysis using maximum likelihood and oblique rotation was conducted. Inspection of the scree plot, eigenvalues, and minimum average partial correlation were used to determine the optimal factor structure to extract.

**Results:**

A total of 498 people with BD (349/498, 70.1% female; mean age 39.64, SD 12.54 years; 181/498, 36.3% BD type I; 195/498, 39.2% BD type II) consented to sharing their QoL Tool data for the present study. Mean scores across the 14 QoL Tool domains were, in general, significantly lower than that of the original QoL.BD validation sample. Reliability estimates for QoL Tool domains were comparable with that observed for the QoL.BD instrument (Cronbach alpha=.70-.93). Exploratory factor analysis supported the extraction of an 11-factor model, with item loadings consistent with the factor structure suggested by the original study. Findings for the sleep and physical domains differed from the original study, with this analysis suggesting one shared latent construct.

**Conclusions:**

The psychometric properties of the web-based QoL Tool are largely concordant with the original pen-and-paper QoL.BD, although some minor differences in the structure of the sleep and physical domains were observed. Despite this small variation from the factor structure identified in the QoL.BD instrument, the latent factor structure of the QoL Tool largely reproduced the original findings and theoretical structure of QoL areas relevant to people with BD. These findings underscore the research and clinical utility of this instrument, but further comparison of the psychometric properties of the QoL Tool relative to the QoL.BD instrument is warranted. Future adaptations of the QoL Tool, including the production of an app-based version of the QoL Tool, are also discussed.

## Introduction

### Background

Applications of quality of life (QoL) assessment instruments in bipolar disorder (BD) research have grown substantially [1,2] since the introduction of the concept in psychiatric research more generally in the 1980s [3]. Broadly speaking, QoL instruments holistically assess an individual’s satisfaction and functioning across a range of life domains and are, therefore, increasingly used to evaluate BD treatment outcomes beyond symptomatic response. Assessment of QoL may be particularly important in the context of BD, given the chronic course and significant impacts across diverse life domains associated with this mood disorder [1]. Indeed, there is some evidence to indicate that both patients with BD and health care providers view QoL as the most important outcome in the treatment of the condition [4].

In the study of BD, QoL has been primarily measured with universal or generic instruments (most commonly, the 36-Item Short Form Health Survey and Quality of Life Enjoyment and Satisfaction Questionnaire [1]). Although generic measures assess areas of life, which may be considered fundamentally important [5], patient groups may have unique priorities that are best assessed with disorder-specific instruments [6]. To address this gap, the first condition-specific QoL instrument for BD was developed: the Quality of Life in Bipolar Disorder (QoL.BD) [7]. Informed by consultation with people with lived experience of BD, their family members, and field experts, the resulting scale assesses cardinal life areas directly impacted by BD symptoms (mood, sleep, physical health, and cognition), pragmatic and functional outcomes (home, work, education, leisure, and finances), and more psychosocially orientated constructs (relationships, self-esteem, spirituality, identity, and independence). A decade since its development, the QoL.BD instrument has seen international adoption: it has undergone formal adaptation and validation in Iranian, Chinese, and Chilean populations [8-10] and has been translated into over 20 languages [11]. It has also seen application in diverse research contexts, including clinical trials of psychotherapy [12-16] and pharmacological interventions [17,18]. Materials have also been developed to support the use of the QoL.BD instrument by health care practitioners in a clinical context (eg, case formulation [11]) and by individuals with BD themselves in their self-management practices [19].

Although the uptake of the QoL.BD instrument has been encouraging, its research and clinical utility may be enhanced with a web-based delivery format. Relative to traditional pen-and-paper instruments, web-based administration formats reduce administrative burden (through, for instance, automatic scoring and practical data storage), data entry and coding errors, and item nonresponse [20]. Web-based questionnaires may also enhance the accessibility of instruments for both researchers and patients: they are cost-effective [21], instantaneously available to potential users with an internet connection (regardless of location), and navigation is user-friendly, with the ability to skip or eliminate irrelevant questions from the view [22]. Respondents may also prefer web-based administration formats [23], and for questionnaires that assess sensitive topics (such as factors related to mental health), web-based questionnaires may potentially reduce social desirability effects [24]. For ongoing self-monitoring purposes, web-based instruments are advantaged by their ability to provide immediate feedback to respondents, reduce the burden of tracking large volumes of data, and potentially lessen experiences of stigma by decreasing the visibility of symptom monitoring. Given the numerous pragmatic benefits and enhanced user-friendliness of web-based self-report questionnaires, adaptation of the pen-and-paper version of the QoL.BD instrument to a web-based interface was undertaken to support utilization of this instrument across research, clinical, and self-management contexts.

However, simple migration of pen-and-paper scales to web-based formats does not guarantee preservation of a scale’s psychometric properties. A number of factors can impact the way a scale performs when adapted for web-based administration, including modifications to layout, instructions, or changes in item wording and response options [25,26]. The Professional Society for Health Economics and Outcomes Research (ISPOR) guidelines suggest that evidence needed to support measurement equivalence between pen-and-paper and electronic adaptations varies depending on the extent of modifications, from minor (eg, simply displaying a scale text on screen) to more substantive (ranging from moderate alterations such as splitting the presentation of items over several screens, up to large scale changes to items, presentation or response format). Supporting evidence can include usability testing, appraisal of interformat reliabilities, comparable means and standard deviations, and preservation of scale reliability and factor structures across formats. Although the majority of Web-adaptation studies have reported interformat reliabilities, informing confidence about the consistency of measurement of self-reported mental health data across formats [27,28], fewer studies have made a comment on whether the original factor structure is replicated in web-based questionnaire formats. As such, exploration of the psychometric properties, particularly factor structure, is needed to support the use of any web-based adaptation of the QoL.BD instrument.

### Objective

The overarching aim of this study was to compare the psychometric performance of the web-based QoL Tool with the original pen-and-paper version of the QoL.BD scale. To do this, we aimed to (1) describe the means and standard deviations of QoL Tool responses, (2) evaluate the internal consistency of the web-based measure, and (3) determine whether the factor structure of the original QoL.BD could be replicated in the web-based adaptation of this instrument.

## Methods

### Overview

The project was conducted by the Collaborative RESearch Team to study psychosocial issues in Bipolar Disorder (CREST.BD [19]), a Canadian-based network dedicated to collaborative research and knowledge translation (KT) in BD. CREST.BD specializes in community-based participatory research (CBPR), where researchers and knowledge users work collaboratively [29]. Informed by a decade of research and integrated KT, CREST.BD has developed a specific model of CBPR for BD [30]. Funding from the Canadian Institutes of Health Research was granted to extend on prior work to design and validate a pen-and-paper QoL questionnaire for BD (described below). This psychometric evaluation follows the development of a web-based adaptation of the QoL.BD instrument using CBPR methods.

### Design and Validation of the Pen-and-Paper Quality of Life in Bipolar Disorder Questionnaire

The development and validation of the QoL.BD instrument is described in detail elsewhere [7]. In brief, candidate items were generated through (1) qualitative interviews with people with BD, their family members, and field experts and (2) a literature review of existing research on QoL in BD. Following item reduction, preliminary psychometric analyses, and further consultation with field and lived experience experts, a final subset of 56 items was retained. Items are organized into 14 4-item domains: 12 core (physical, sleep, mood, cognitive, leisure, social, spirituality, finances, household, self-esteem, independence, and identity) and 2 optional (work and study, which respondents are directed to complete if they are currently employed or in school). A 12-item brief version was also developed.

The questionnaire items are presented on a standard 5-point Likert response scale (strongly disagree–strongly agree). Items are all positively worded (ie, describing the presence of a desirably quality) for two reasons: (1) a positive question frame is consistent with the strengths-based approach to QoL adopted as a result of CBPR consultation and (2) reverse worded items can reduce the reliability and validity of a scale [31]. Domains are scored by summing the responses, for a potential score range of 4 to 16. Calculation of an overall QoL score is possible by summing responses to the 12 core domains. Initial field testing of the QoL.BD instrument indicated that both the full and brief version of the instrument represent a feasible, reliable, and valid BD-specific QoL measure with solid internal validity and appropriate test-retest reliability. Factor analysis affirmed that the 12 basic scales were represented in the latent structure of the instrument.

### Development of the Web-Based Adaptation: The Quality of Life Tool

A synergistic combination of CBPR and the principles of user-centered design [32] were applied to develop the QoL Tool. The primary goal of the development process for the QoL Tool was to produce a web-based version of the QoL.BD instrument that was faithful as possible to the original measurement principles of the QoL.BD instrument but also adapted and expanded to enhance user experience and functionality. A priori, we established which features of the QoL.BD instrument were immutable, specifically, preservation of precise wording of the scale’s 56 items (with one exception, described below), the ordering of the items, and the 5-point Likert response scale (1=strongly disagree; 5=strongly agree) and item ordering. The approach to scoring the domains and the range of potential scores are consistent with the QoL.BD instrument.

**Figure 1:**
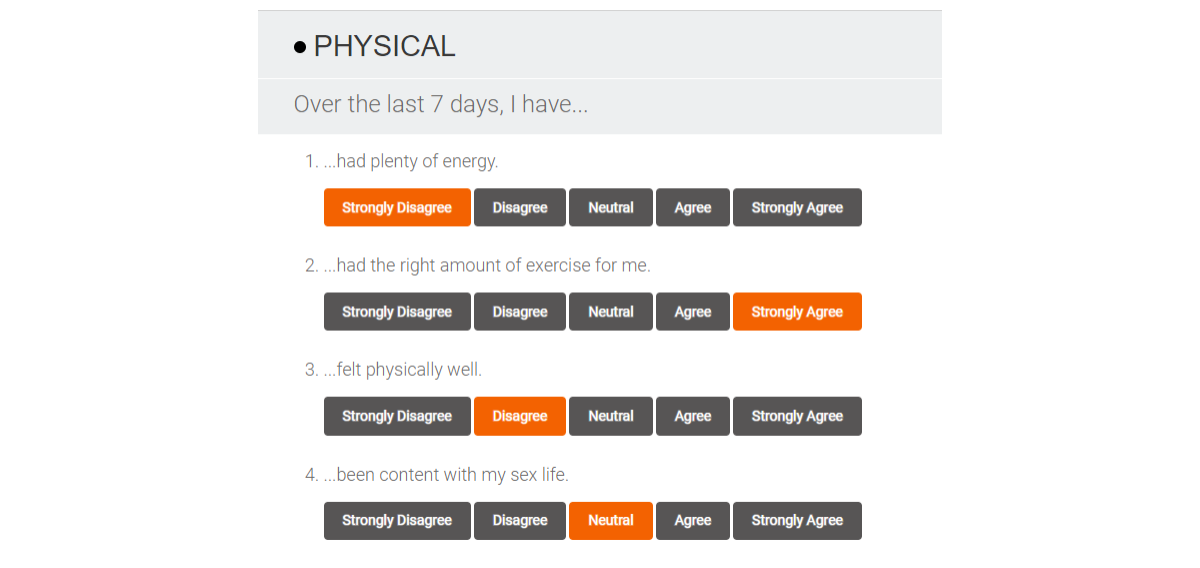
QoL Tool questionnaire screen and response options.

**Figure 2:**
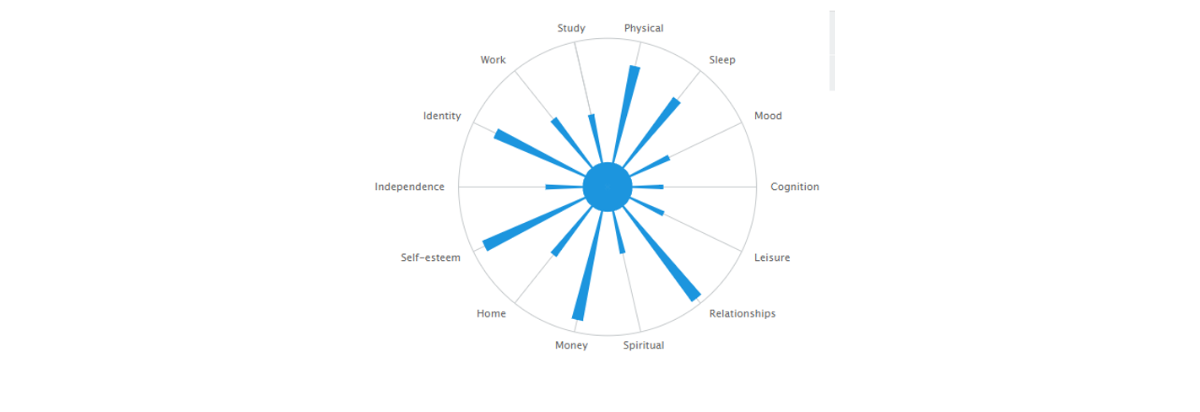
QoL Tool graphical display of results.

**Figure 3:**
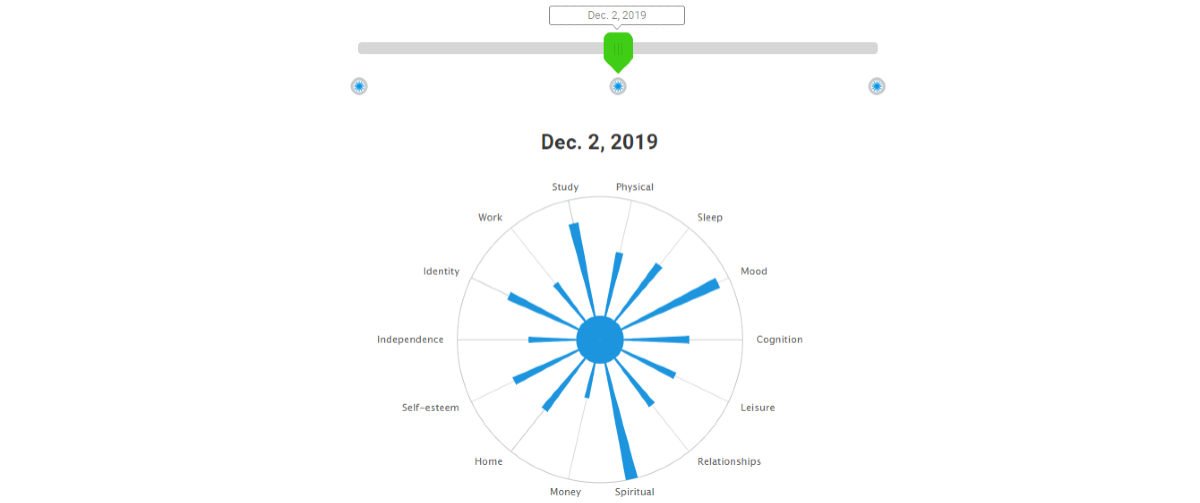
Users are able to drag a slider to compare their QoL Tool results over time.

Beyond these parameters, however, it was expected that the web-based version of the scale would differ in some aspects from its pen-and-paper counterpart. One adaptation was made in the delivery format of the web-based version on the basis of user feedback, the name of each domain was made visible to the user (see Figure 1). Furthermore, as the inclusion of graphical feedback of results has been described as a highly prioritized feature for self-management apps for people with BD [32,33], we determined *a priori* that the addition of a results display feature would be essential (see Figure 2). All other adaptations were identified via user-centered design processes. One minor change in wording was made to a sleep domain item (“woken up” was changed to “awoken”). All other items in the QoL Tool were precisely as worded in the QoL.BD instrument. Substantial changes were made in the web-based version in terms of features and functionality. For example, registered users of the QoL Tool are provided with the option of an interactive results feature that demonstrates their QoL scores over time (Figure 3) and the option to email their results to a health care provider.

### Recruitment

The final version of the QoL Tool was formally launched on World Bipolar Day (March 30, 2015). The QoL Tool was promoted primarily through social media channels (eg, CREST.BD Facebook page, Twitter, and website) but was also highlighted as part of a series of knowledge translation events (May-June 2015) focused on sharing knowledge on self-management strategies for BD [34]. Informed consent for data collection for research purposes was provided at the point of registration in the QoL Tool system but was not required for the use of the web-based interface. Inclusion criteria were (1) older than 19 years; (2) have a self-reported diagnosis of BD I, II, or not otherwise specified (NOS); and (3) able to communicate in English. No compensation was offered for participants’ completion of the QoL Tool. Ethics approval was obtained from the University of British Columbia’s Behavior Research Ethics Board.

### Data Collection

Data collection for this study occurred between March 30, 2015, and October 17, 2018. Demographic details and responses to the QoL Tool were saved in a secure database hosted at the University of British Columbia. For the purposes of this study, only baseline responses to the QoL Tool were analyzed to avoid contaminating the extracted factor structure with potential learning effects [35]. Data from the baseline time point of the original QoL.BD validation study (n=224; sample described [7]) were included where relevant for comparison purposes.

### Statistical Analysis

#### Internal Consistency

Internal consistency was evaluated using Cronbach alpha for each of the 14 domains. Scales were deemed to be adequately reliable if Cronbach alpha exceeded .7 [36].

#### Exploratory Factor Analysis

Analyses were carried out on the 12 basic domains of the web-based QoL.BD ie, all except work and study). Factorability was confirmed by using the Kaiser-Meyer-Oklin measure of sampling adequacy and Bartlett test of sphericity. No corrections for missing data were required, as the design of the QoL Tool does not permit users to submit their data unless responses have been provided for all questions. An exploratory factor analysis (EFA) using maximum likelihood extraction was conducted in SPSS 27 (IBM, Armonk, New York). Given that the variables were assumed to be correlated, oblique (oblimin) rotation was applied. Multiple criteria were reviewed to determine the optimal number of factors retained. First, visual inspection of the point of inflexion displayed by the scree plot was conducted [37]. Second, Kaiser criterion was used to determine the number of factors with eigenvalues with a value greater than 1 [38]. Third, the minimum average partial correlation (MAP) test was applied using SPSS [39] to identify the number of components that produces the minimum mean squared partial correlation [40]. Finally, the percentage of variance explained by each factor was considered: amount of variance explained (with 5% a generally accepted cutoff) may be used as a decision rule [41,42].

Interpretability of the extracted factors was evaluated by confirming primary factor loadings exceeded 0.4 and that cross-loadings were less than 0.3 [42]. Finally, the item content of each factor was evaluated to confirm whether the domains proposed by the original validation of the QoL.BD instrument were represented.

## Results

### Participants

A final sample of 498 participants (349/498, 70.1% female; 128/498, 25.7% male; 6/498, 1.2% transgender or nonbinary) with a mean age of 39.64 years (SD 12.54) were included in the analysis. In total, 36.3% (181/498) of the participants reported having a diagnosis of BD type I (BD-I), 39.2% (195/498) reported having a diagnosis of BD type II (BD-II), and 2.2% (11/498) self-identified as having a diagnosis of BD NOS. Individuals with other unspecified BD (50/498, 10.5%), unclear or pending diagnoses (31/498, 6.5%), or rapid-cycling BD (7/498, 1.5%) comprised the remainder. The majority of participants were located in North America (22/498, 44.2% Canadian; 138/498, 27.7% American), with the remainder comprising international respondents (most commonly from Australia with 28/498, 5.6% or Germany with 16/498, 3.2%). Characteristics of this sample (QoL Tool respondents) and the original pen-and-paper QoL.BD validation sample can be found in Table 1. The two samples did not significantly differ with respect to gender composition, X^2^_1_=1.6 (N=690), *P*=.21; nor age, *t*_720_=1.31, *P*=.19. The two samples did differ with respect to diagnosis, X^2^_1_=53.7 (N=590), *P*<.001; with the QoL Tool sample containing a greater proportion of individuals with BD-II than the pen-and-paper sample.

**Table 1:** Sample characteristics of the quality of life (QoL) Tool (n=498) and Quality of Life in Bipolar Disorder (n=224) validation sample.

Sample characteristics		QoL Tool validation sample (n=498)	Original Quality of Life in Bipolar Disorder validation sample (n=224)
**Gender, n (%)**			
	Male	128 (25.7)	67 (29.9)
	Female	349 (70.1)	146 (65.2)
	Trans or nonbinary	6 (1.2)	N/A^a^
Age (years), mean (SD)		39.64 (12.54)	41.00 (13.67)
**Diagnosis, n (%)**			
	BD-I^b^	181 (36.3)	169 (75.4)
	BD-II^c^	195 (39.2)	45 (20.1)
**Work/employment status, n (%)**			
	Currently engaged in paid or volunteer employment	317 (63.7)	121 (54.0)
	In education	154 (30.9)	52 (23.2)

^a^N/A: not applicable.

^b^BD-I: bipolar disorder type I.

^c^BD-II: bipolar disorder type II.

### Distributions by Domain

Mean, standard deviation, and skew of the 14 domains of the QoL Tool are presented in Table 2, along with comparison data from the first time point of the original QoL.BD validation study. The optional work and study sections were completed by 63.7% (317/498) and 30.9% (154/498) of the web-based sample and 54% (121/225) and 23.2% (52/224) of the pen-and-paper sample, respectively. The distribution of QoL Tool domain scores was approximately normal, with all skew values well under the recommended absolute value of 2 [43] and no evidence of floor or ceiling effects. Mean scores for the web-based sample were significantly lower across all domains except finance, relative to the pen-and-paper sample.

**Table 2:** Descriptive statistics for the 12 basic and two optional domains of the quality of life (QoL) Tool.

Domain	QoL Tool		Quality of life in Bipolar Disorder			*t* test value (*df*)		*P* value	
	Mean (SD)	Skew		Mean (SD)	Skew				
Physical	9.61 (3.70)	0.37		11.77 (4.01)	0.01		7.27 (740)		<.001
Sleep	10.05 (3.64)	0.25		11.42 (4.12)	0.04		5.79 (740)		<.001
Mood	10.74 (3.89)	0.05		12.98 (3.98)	−0.27		7.31 (740)		<.001
Cognitive	10.91 (3.68)	0.06		12.78 (4.22)	−0.26		6.19 (740)		<.001
Leisure	11.79 (3.99)	−0.17		13.11 (4.16)	−0.24		4.17 (740)		<.001
Social	12.83 (4.12)	−0.36		14.31 (4.00)	−0.67		4.64 (740)		<.001
Spirituality	11.49 (3.97)	−0.05		13.05 (4.07)	−0.39		4.99 (740)		<.001
Finances	12.42 (4.89)	−0.13		12.67 (4.64)	−0.28		0.67 (740)		.51
Household	10.91 (4.27)	0.06		12.96 (4.11)	−0.32		6.22 (740)		<.001
Self-esteem	12.90 (3.64)	−0.35		13.82 (3.70)	−0.44		3.22 (740)		.001
Independence	14.51 (3.35)	−0.70		15.78 (3.23)	−0.84		4.91 (740)		<.001
Identity	11.15 (4.10)	0.07		13.68 (4.14)	−0.25		7.87 (740)		<.001
Work (optional)	12.83 (4.25)	−0.32		15.20 (3.52)	−0.96		5.46 (436)		<.001
Study (optional)	11.56 (4.26)	−0.02		13.92 (4.96)	−0.48		3.31 (204)		.01

### Internal Consistency

Acceptable to excellent reliability estimates were observed for all 14 QoL Tool domains (see Table 3 for Cronbach alpha values for the QoL Tool and comparison data from the first time point of the original QoL.BD validation study). Reliability estimates for the QoL Tool were comparable with those reported for the QoL.BD instrument across all domains.

**Table 3:** Internal consistency estimates for the quality of life (QoL) Tool and Quality of Life in Bipolar Disorder.

Domain	QoL Tool		Quality of Life in Bipolar Disorder	
	Cronbach alpha	n	Cronbach alpha	n
Physical	.70	498	.79	218
Sleep	.77	498	.83	208
Mood	.88	498	.90	220
Cognitive	.83	498	.91	219
Leisure	.89	498	.91	193
Social	.86	498	.88	220
Spirituality	.91	498	.93	214
Finances	.89	498	.88	214
Household	.93	498	.91	217
Self-esteem	.84	498	.88	221
Independence	.76	498	.81	217
Identity	.86	498	.90	220
*Work*	.90	317	.89	121
*Study*	.89	154	.95	52

### Exploratory Factor Analysis

The suitability of the data for factor analysis was confirmed, with a “very good” sample size [44] and appropriate factorability. The Bartlett test of sphericity was significant, c^2^_1128_=15,621.64; *P*<.001, and the Kaiser-Meyer-Olkin measure of sampling adequacy was .93, exceeding the recommended minimum value of .60 [35].

Visual inspection of the scree plot using the William guidelines [37] suggested an 11-factor structure. The Kaiser criterion suggested an 11-factor structure, which accounted for 70.48% of the variance. The MAP test identified that the extraction of 12 components was required to produce the minimum mean squared partial correlation. Weighting these findings together, both an 11-factor structure and a 12-factor structure were considered. Owing to the fact that retention of the 12th factor explained less than 2% of the additional variance (below the 5% cutoff used for factor retention [41]), a final 11-item factor structure was retained.

The interpretability of the 11 extracted factors was supported, with the majority of factors (n=9) having at least four items with factor loadings above 0.4. Only one item (“I have felt emotionally balanced”) was observed to have a cross-loading above 0.3 (primary loading mood, secondary loading cognition). The pattern matrix with oblique rotation and factor loadings above 0.3 is shown in Multimedia Appendix 1. The item content of the extracted factors largely aligned with the factor structure suggested in the original validation study, as well as the conceptual labeling of the domains, with the exception of items belonging to the sleep and physical domains. The rotated factor structure suggested that a single latent factor best explained the variance of items belonging to these domains. Furthermore, two items from the original physical domain (“I have had the right amount of exercise for me” and “I have been content with my sex life”) did not demonstrate significant loadings on any extracted factor.

## Discussion

### Principal Findings

This study describes the psychometric properties of a web-based QoL questionnaire for individuals with BD (the QoL Tool) adapted from a pen-and-paper measure (QoL.BD [7]). Distributions of the core 12 QoL Tool domains were comparable with those found using the QoL.BD instrument in the original validation sample (although QoL Tool respondents reported lower QoL across the majority of domains), standards for appropriate internal consistency were met, and EFA suggested a factor structure that is adequately concordant with the full pen-and-paper version.

EFA of the core item set of the QoL Tool suggested an 11-factor latent structure, accounting for a similar proportion of variance (70.48%) to the factor structure identified in the original QoL.BD validation study (12-factor structure accounting for 71% of variance). Furthermore, the same items (with the exception of certain sleep and physical items) were observed to load on the domains identified by the original validation study and conceptual structure of the QoL.BD instrument, suggesting that the same constructs are being measured by the web-based and paper-based versions of this instrument [35].

A range of data suggests that, not surprisingly, directly copying a paper questionnaire into a web page results in negligible change to its psychometric properties. A systematic review conducted by Alfonsson et al [27] on the adaptation of 40 symptom scales into digital format indicated that most web-based instruments appear reliable across administration formats. More specifically, van Ballegooijen et al [28] conducted a focused review of the psychometric data of digitized paper questionnaires measuring symptoms of mood and anxiety disorders, demonstrating adequate psychometric properties of the tools in their web-based formats. Despite a growing body of evidence comparing the psychometric properties of web-based adaptations of questionnaires, three notable limitations of this body of work exist. First, studies have typically examined the interformat reliability (Web-based vs pen-and-paper) of psychosocial instruments. Second, those which have validated web-based mental health measures have typically used general population samples rather than testing the instrument in a clinical population. Third, few studies have reported the type and extent of modifications made in adapting pen-and-paper questionnaires to a web-based format [27], limiting the ability to make inferences about the effect of delivery mode on degree of similarity. Consequently, this study contributes some initial evidence (couched in the limitations discussed below) supporting that factor structure may be largely preserved in web-based adaptations of patient-reported outcomes including minor to moderate modifications (as defined by ISPOR recommendations [25]) when tested in a BD sample.

Although the EFA results support that, overall, the QoL Tool and QoL BD have concordant factor structures, one point of divergence warrants further discussion. In the QoL Tool sample, EFA results suggest one latent factor may best account for items from both the sleep and physical QoL domains, whereas the original validation study of the QoL.BD instrument supported two distinct factors. This design does not allow us to unpack the determinant of this difference. We can speculate that, potentially, the minor-to-moderate modifications in the web-based delivery of the QoL Tool (changes to wording of one item, a graphical representation of results, and the ability for users to see the domain names) may have contributed to this small divergence in factor structure. A second candidate explanation that must be considered is differing sample compositions [45]: In this study, similar proportions of individuals reporting BD-I and BD-II diagnoses participated; the original validation study predominantly consisted of individuals with BD-I (see Table 1). Given that the prevalence of various physical health comorbidities [46,47] and the experience of sleep disturbances [48] may vary according to BD subtype, heterogeneity between samples may underpin the differences in factor structure observed over the sleep and physical items viz the original validation study.

There are no clear-cut guidelines regarding the optimal way to respond when faced with points of divergence in the psychometric properties of web-based and pen-and-paper instruments; developers must consider the impact on psychometric properties in light of the numerous advantages of web-based adaptations (discussed above) as well as supporting interpretation of results by preserving the surface structure and face validity of the questionnaire. ISPOR recommendations highlight that fidelity to the original pen-and-paper instrument must be balanced against the potential to improve functionality and performance in web-based adaptations [25], and as such, concrete, universal recommendations about standards of evidence and quantitative cutoffs for acceptable psychometric properties cannot be made. In fact, the meaning of divergence between pen-and-paper instruments and web-based adaptations is not clear cut and may in fact reflect *improved* data quality and user-friendliness on the part of the web-based instrument, as the potential for social desirability effects or missing data to bias findings is ameliorated. Furthermore, psychometric findings about factor structure is only one piece of evidence which should drive decisions about the surface structure of an instrument. In the case of this study, although it is perhaps unsurprising that a latent factor may underpin items assessing both sleep and physical health, given that some of the physical health items (eg, “I have had plenty of energy”) are likely to be impacted by achieving adequate sleep, there is also evidence to suggest the face validity of distinct domains. Assessing these domains separately is key for the instrument to have clinical and research utility, given that sleep changes in BD are one of the most prominent prognostic indicators of mood destabilization [49], and sleep difficulties require different self-management and clinical interventions relative to physical health comorbidities [50]. In light of this and given strong conceptual arguments for separate sleep and physical domains, we suggest these items continue to be scored according to the original QoL.BD.

### Limitations

A number of limitations to this study should be noted. First, participants self-reported their diagnosis of BD; although the confirmation of diagnosis by structured psychiatric interview would have been preferable, there is some evidence that people who self-identify as living with BD typically do meet diagnostic criteria [51]. Furthermore, as the sample was self-selected, higher levels of digital literacy may have been present: qualitative interviews with a small subsample of participants who were given the opportunity to use a web-based BD self-management intervention suggest that some participants struggled to access that website because of technological barriers [34]. Care must be taken to evaluate the feasibility and psychometric properties of the QoL Tool in samples with lower levels of digital literacy or those facing a digital divide.

Finally, the web-based and pen-and-paper versions of the QoL.BD instrument were not directly compared in the same sample. Therefore, we are unable to determine whether factor equivalence was impacted by differing demographic compositions, rather than modifications to the delivery of the instrument itself. Furthermore, we were not able to assess concordance with the pen-and-paper QoL.BD in the form of intraclass correlation coefficients. However, it has been noted that it is not typically feasible nor warranted to assess test-retest reliability across instrument modes [25] and indeed, this may introduce confounding learning effects.

### Implications and Future Directions

This study provides evidence for concordance between the web-based and paper-based versions of the widely adopted QoL.BD questionnaire, providing some confidence in the use of the QoL Tool in research or clinical assessments. Furthermore, there is now also qualitative evidence to suggest that the QoL Tool can be integrated positively into the self-management practices of individuals with BD; respondents described the breadth of areas assessed as enabling them to identify areas of strengths as well as areas in need of improvement [52]. This emerging body of evidence for the utility of the QoL Tool, both from a psychometric and subjective perspective, suggests further development and dissemination of this measure is warranted.

One avenue of expansion is the translation of the QoL Tool from a web-based interface to a mobile phone app. The project to develop the QoL Tool was initiated in 2014; significant advances have occurred in the digital mental health landscape since that time—we now need to avail of developing technologies to enhance the delivery and functionality of this instrument. People with BD have shown interest in digitally supported self-management [32,53], and self-monitoring apps have been found to be feasible and acceptable in this population [54-56]. However, current apps do not adequately meet consumer needs to track a broad spectrum of outcomes [32], with most apps developed to assess domains of well-being in isolation, such as sleep or mood. Individuals with BD have described resorting to elaborate, self-generated systems to track multiple indicators [57]; between-app integration is a requested feature of apps for BD [32]. The range of wellness outcomes assessed by the QoL Tool means that its adaptation into an app format is likely to meet this consumer need.

Moving forward, CREST.BD has now initiated a 3-year project to incorporate the evidence and tools held in the Bipolar Wellness Centre [58] and the QoL Tool [19] into a new mobile health app—“Bipolar Bridges.” The project aims to address some of the limitations of existing BD apps by using CBPR approaches to co-design an app that synergistically combines different forms of digital health data (including QoL Tool results), enabling individuals to learn what self-management strategies are most effective for optimizing their QoL.

### Conclusions

This study provides initial support for the psychometric validity of the QoL Tool, a web-based adaptation of an instrument to measure QoL in BD (the QoL.BD instrument). Specifically, internal consistency estimates, distribution of scores, and factor structure were largely consistent with the pen-and-paper version. Although evidence supporting the overall equivalence of these instruments was observed, the findings of this study suggested a latent structure of 11 compared with the original 12 basic domains in the original instrument. This 11-factor structure combined items from the sleep and physical domains in a single shared factor. Two explanations for this minor divergence must be considered: changes to the user experience in the web-based interface and differences in sample composition. As the design of this study does not permit separating the influence of these two potential explanations, further research is required. However, in light of the face validity and clinical utility of a distinct sleep domain in QoL in BD, we recommend these items continue to be treated according to the structure of the original QoL.BD. Given the increasing role of web-based self-report questionnaires for research, clinical, and self-management contexts, findings of overall psychometric equivalence between these QoL instruments validate current applications of the QoL Tool and encourage further efforts to optimize its web-based delivery and associated self-management strategies via a novel mobile phone app.

## Appendix

Multimedia Appendix 1 Primary factor loadings of the quality of life tool based on an exploratory factor analysis with maximum likelihood extraction and oblique rotation.

## Abbreviations

BD: bipolar disorder
BD-I: bipolar disorder type 1
BD-II: bipolar disorder type 2
CBPR: community-based participatory research
CREST.BD: The Collaborative RESearch Team to study psychosocial issues in Bipolar Disorder
EFA: exploratory factor analysis
ISPOR: Professional Society for Health Economics and Outcomes Research
KT: knowledge translation
MAP: minimum average partial correlation
NOS: not otherwise specified
QoL.BD: Quality of Life in Bipolar Disorder
QoL: quality of life
