# Supplementary material for: A Web-Based Adaptation of the Quality of Life in Bipolar Disorder Questionnaire: Psychometric Evaluation Study
Source: JMIR Ment Health. 2020 Apr 27;7(4):e17497. doi: 10.2196/17497 (PMC7215515; doi:10.2196/17497)
Supplement: Multimedia Appendix 1 [file mental_v7i4e17497_app1.docx]

Multimedia Appendix 1. Primary factor loadings of the QoL Tool based on an exploratory factor analysis with maximum likelihood extraction and oblique rotation.

| **“Over the past 7 days, I have…”** | **Primary loading** | **Factor** |
| --- | --- | --- |
| Felt happy | 0.57 | 1 (Mood) |
| Enjoyed things as much as I usually do | 0.51 | 1 |
| Felt able to cope | 0.50 | 1 |
| Felt emotionally balanced | 0.40 | 1 |
| Done my daily household chores | 0.83 | 2, (Household) |
| Been organized around my home | 0.89 | 2 |
| Kept my home tidy | 0.95 | 2 |
| Kept my home clean | 0.85 | 2 |
| Had enough money for basic needs | 0.83 | 3, (Finances) |
| Had enough money for extras | 0.92 | 3 |
| Felt secure about my current financial situation | 0.82 | 3 |
| Had no difficulties with debts | 0.73 | 3 |
| Been satisfied with the spiritual side of my life | 0.86 | 4, (Spirituality) |
| Expressed my spirituality as I wish | 0.90 | 4 |
| Practised my spirituality as I wish | 0.92 | 4 |
| Kept routine in my spiritual life | 0.74 | 4 |
| Had a strong sense of self | 0.72 | 5, (Identity) |
| Had a stable sense of what I’m really like | 0.90 | 5 |
| Had a clear idea of what I want and don’t want | 0.63 | 5 |
| Had control over my life | 0.41 | 5 |
| Enjoyed spending time with other people | 0.60 | 6, (Social) |
| Been interested in my social relationships | 0.75 | 6 |
| Had meaningful friendships | 0.74 | 6 |
| Been able to share feelings or problems with a friend | 0.74 | 6 |
| Felt respected | 0.69 | 7, (Self-esteem) |
| Felt accepted by others | 0.82 | 7 |
| Felt as worthwhile as other people | 0.48 | 7 |
| Felt able to cope with stigma | 0.32 | 7 |
| Had plenty of energy | 0.35 | 8, (Sleep / Physical) |
| Had the right amount of exercise for me | - | 8 |
| Felt physically well | 0.33 | 8 |
| Been content with my sex life | - | 8 |
| Woken up feeling refreshed | 0.75 | 8 |
| Had no problems getting out of bed | 0.62 | 8 |
| Had about the right amount of sleep for me | 0.70 | 8 |
| Kept a routine in my sleep‐wake schedule | 0.41 | 8 |
| Thought clearly | 0.68 | 9, (Cognition) |
| Had good concentration | 0.67 | 9 |
| Had no difficulties with my memory | 0.61 | 9 |
| Made plans without difficulty | 0.44 | 9 |
| Had a sense of freedom | 0.45 | 10, (Independence) |
| Felt safe in my home environment | 0.57 | 10 |
| Traveled around freely (e.g., driving, using public transport) | 0.61 | 10 |
| Felt others have allowed me my independence | 0.76 | 10 |
| Enjoyed my leisure activities | -0.87 | 11, (Leisure) |
| Been interested in my leisure activities | -0.84 | 11 |
| Had fun during my leisure activities | -0.80 | 11 |
| Expressed my creativity | -0.34 | 11 |
